# Supplementary material for: Availability of priority maternal and newborn health indicators: Cross-sectional analysis of pregnancy, childbirth and postnatal care registers from 21 countries
Source: PLOS Glob Public Health. 2023 Jan 5;3(1):e0000739. doi: 10.1371/journal.pgph.0000739 (PMC10021477; doi:10.1371/journal.pgph.0000739)
Supplement: S1 File — (DOCX) [file pgph.0000739.s001.docx]

**Core Health Facility Indicators for Maternal Health- ANC, Childbirth and PNC**

| **Core indicators** | **Definition** | **Computation** |
| --- | --- | --- |
|  |  | **(e.g. numerator/denominator, number)** |
| **Maternal health** | | |
| **Antenatal client 1st visit before 12 weeks gestation** | Percentage of antenatal clients with 1st visit before 12 weeks | N: Number of antenatal clients 1st visit before 12 weeks  D: Number of antenatal clients 1st visit |
| **Antenatal care 8th visit** | Percentage of antenatal clients who had 8 ANC visits | N: Number of antental clients with 8th ANC visit  D: Estimated number of pregnant women |
| **Antenatal client syphilis screening** | Percentage of antenatal clients screened for syphilis | N: Number of antenatal clients screened for syphilis D: Number of antenatal clients 1st visit |
| **Antenatal client haemoglobin measured** | Percentage of antenatal clients with haemoglobin level measured | N: Number of antenatal clients with haemoglobin level measured  D: Number of antenatal clients 1st visit |
| **Antenatal client blood pressure measurement** | Percentage of antenatal clients with blood pressure measured | N: Number of antenatal clients with blood pressure measured |
|  |  | D: Number of antenatal clients 1st visit |
| **Prevention of mother- to-child transmission (PMTCT) - testing coverage rate** | See *Analysis and use of health facility data - Guidance for HIV programme managers* for more details on PMTCT; numerator and denominator taken from this document. | N: Number of pregnant women attending ANC and/or who had a facility-based delivery who were tested for HIV during pregnancy or already knew they were HIV-positive  D: Number of ANC attendees or number of facility- based deliveries |
| **Intermittent preventive therapy for malaria during pregnancy (IPTp)** | See Analysis and use of health facility data - Guidance for HIV programme managers for more details on PMTCT; numerator and denominator taken from this document.   More details on coverage of IPTp; numerator and denominator taken from this document. | N: Number of pregnant women given at least three doses of sulfadoxine/purimethamine for IPT  D: Number of antenatal clients 1st visit |
| **Iron supplementation for pregnant women** | See *Collection, analysis and use of health facility and community data – Guidance for nutrition programme managers†* for more details. | *Nutrition guidance document and indicator definitions under development* |
| **Caesarean section** | Percentage of deliveries in health facilities by caesarean section | N: Number of caesarean sections in a facility D: Number of deliveries in facility |
|  |  |  |
| **Uterotonic for prevention of post- partum haemorrhage** | Percentage of women who gave birth in a facility who received a prophylactic uterotonic  (e.g.Oxytocin) immediately after birth for prevention of postpartum hemorrhage «Immediately» ideally refers to within one minute | N: Number of women who gave birth in a facility who received a prophylactic uterotonic immediately after birth  D: Number of deliveries in facility |

**Additional Health Facility Indicators for Maternal Health – PNC and SRH/FP**

| **Core indicators** | **Definition** | **Computation**  **(e.g. numerator/denominator, number)** |
| --- | --- | --- |
| **Postnatal** | | |
| **Notification for birth registration** | See *Collection, analysis and use of health facility and community data: Guidance for health programme managers on vital events data** for more details on notification for birth registration.  Note: In many countries the health system has the mandate to notify births to the civil registry or to provide documentation to parents for registration. | N: Number of babies/ children for whom notifications are issued for birth registration within specified number of days after birth D: Number of live births in facility  Note: The specified number of days after birth should be aligned with national policy/guidelines. |
| **Babies with documented birthweight** | Percentage of babies born in a facility with documented birthweight before discharge | N: Number of babies born in a facility with documented birthweight before discharge  D: Number of live births in facility |
| **Low-birth weight** | See *Collection, analysis and use of health facility and community data – Guidance for nutrition programme managers†* for more details on low-birth  weight | *Nutrition guidance document and indicator definitions under development* |
| **Newborns breastfed within one hour of birth** | See *Collection, analysis and use of health facility and community data – Guidance for nutrition programme managers†* for more details on  immediate breastfeeding | *Nutrition guidance document and indicator definitions under development* |
| **Postnatal care for women** | Percentage of women with postnatal care (PNC)  Note: The numerator includes both women who gave birth in the health facility and those who gave birth outside the health facility | N: Number of women with postnatal care D: Number of deliveries in facility |
| **Postnatal care for newborns** | Percentage of newborns with postnatal care (PNC)  Note: The numerator includes both newborns who were born in the health facility and those who were born outside the health facility | N: Number of newborns with postnatal care D: Number of live births in facility |
| **Sexual and Reproductive Health** | | |
| **Couple year protection (CYP)** | The estimated protection provided by family planning (FP) services based upon the volume of all contraceptives distributed among the female population 15-49 years. *See notes and example on CYP in Table 1* | The CYP is calculated by multiplying the quantity of each method distributed by a conversion factor, to yield an estimate of the duration of contraceptive protection provided per unit of that method. The CYPs for each method are then summed over all methods to obtain a total CYP figure |
| **Cervical cancer screening** | Percentage of women of reproductive age (15 to 49 years) who were screened for cervical cancer using any of the following methods: visual Inspection with acetic acid/vinegar (VIA), pap smear, human papilloma virus (HPV) test. | N: Number of women of reproductive age who were screed for cervical cancer  D: Estimated number of women of reproductive age |

**Mortality, Malnutrition and TB Health Indicators**

| **Core indicators** | **Definition** | **Computation**  **(e.g. numerator/denominator, number)** |
| --- | --- | --- |
| **Mortality** | | |
| **Maternal deaths in health facility** | Number of women who die in the health facility either while pregnant or within the first 42 days of the end of pregnancy  Note: This can include women who gave birth outside a facility but who died in the health facility. | Number of maternal deaths in facility |
| **Neonatal deaths in health facility** | Number of newborns who die in the health facility in the first 28 days  Note: This includes any neonatal death in a facility that occurred in the first 28 days – pre-discharge  after birth or upon re-admission for an illness. | Number of neonatal deaths in facility |
| **Child deaths in health facility** | Number of children who die in the health facility  Note: This includes deaths that occur between the ages of 1 month up to 9 years of age. | Number of child deaths in facility |
| **Adolescent deaths in health facility** | Number of adolescents who die in the health facility  Note: This includes deaths that occur between the ages of 10 to 19 years of age | Number of adolescent deaths in facility |
| **Stillbirths in health facility** | Stillbirth as a percentage of all births in health facilities  (Baby born with no sign of life and weighing at least 1000g or after 28 weeks gestation) | N: Number of stillbirths in facility  D: Number of live births and stillbirths in facility |
| **Maternal deaths reviewed** | Percentage of maternal deaths reviewed | N: Number of maternal deaths in facility that were reviewed  D: Number of maternal deaths in facility |
| **Perinatal deaths reviewed** | Percentage of perinatal deaths reviewed  Note: Perinatal deaths include stillbirths and newborn deaths up to 7 days after birth | N: Number of perinatal deaths in facility that were reviewed  D: Number of perinatal deaths in facility |
| **Tuberculosis notification** | See [*Analysis and use of health facility data - Guidance for tuberculosis programme managers*](https://www.who.int/healthinfo/FacilityAnalysisGuide_TB.pdf?ua=1) for more details on TB indicators. | Number of tuberculosis cases among children notified in a specified time period, usually one year |
| **Malnutrition** | See *Collection, analysis and use of health facility and community data – Guidance for nutrition programme managers†* for more details on childhood malnutrition. | *Nutrition guidance document and indicator definitions under development* |

**11 Core Indicators for Women and Children’s Health**

| **Indicator** | **Main source** | **Preferred situation** |
| --- | --- | --- |
| Maternal mortality ratio | Surveys | Vital registration |
| Under-five child mortality, with the proportion of newborn deaths | Surveys | Vital registration |
| Children under five who are stunted | Surveys | Surveys |
| Proportion of demand for family planning satisfied (met need for contraception) | Surveys | Surveys |
| Antenatal care coverage (at least four times during pregnancy) | Surveys | Surveys and facility reports |
| Antiretroviral (ARV) prophylaxis among HIV- positive pregnant women to prevent HIV transmission and antiretroviral therapy for [pregnant] women who are treatment-eligible | Facility reports | Facility reports |
| Skilled attendant at birth | Surveys | Surveys and facility reports |
| Postnatal care for mothers and babies within two days of birth | Surveys | Surveys and facility reports |
| Exclusive breastfeeding for six months (0–5 months) | Surveys | Surveys |
| Three doses of combined diphtheria-tetanus- | Surveys and | Surveys and |
| pertussis (DTP3) immunization coverage (12–23 months) | facility reports | facility reports |
| Antibiotic treatment for suspected pneumonia | Surveys | Surveys and facility reports |
